# Study of marine microorganism metabolites: new resources for bioactive natural products

**DOI:** 10.3389/fmicb.2023.1285902

**Published:** 2024-01-08

**Authors:** Noora Barzkar, Stanislav Sukhikh, Olga Babich

**Affiliations:** ^1^Department of Agro-Industrial Technology, Faculty of Applied Science, Food and Agro-Industrial Research Center, King Mongkut’s University of Technology North Bangkok, Bangkok, Thailand; ^2^Research and Education Center “Industrial Biotechnologies”, Immanuel Kant Baltic Federal University, Kaliningrad, Russia

**Keywords:** marine bacteria and fungi, metabolites for disease prevention and treatment, biological activity, bioactive compounds, human health

## Abstract

The marine environment has remained a source of novel biological molecules with diversified applications. The ecological and biological diversity, along with a unique physical environment, have provided the evolutionary advantage to the plant, animals and microbial species thriving in the marine ecosystem. In light of the fact that marine microorganisms frequently interact symbiotically or mutualistically with higher species including corals, fish, sponges, and algae, this paper intends to examine the potential of marine microorganisms as a niche for marine bacteria. This review aims to analyze and summarize modern literature data on the biotechnological potential of marine fungi and bacteria as producers of a wide range of practically valuable products (surfactants, glyco-and lipopeptides, exopolysaccharides, enzymes, and metabolites with different biological activities: antimicrobial, antitumor, and cytotoxic). Hence, the study on bioactive secondary metabolites from marine microorganisms is the need of the hour. The scientific novelty of the study lies in the fact that for the first time, the data on new resources for obtaining biologically active natural products — metabolites of marine bacteria and fungi — were generalized. The review investigates the various kinds of natural products derived from marine microorganisms, specifically focusing on marine bacteria and fungi as a valuable source for new natural products. It provides a summary of the data regarding the antibacterial, antimalarial, anticarcinogenic, antibiofilm, and anti-inflammatory effects demonstrated by marine microorganisms. There is currently a great need for scientific and applied research on bioactive secondary metabolites of marine microorganisms from the standpoint of human and animal health.

## 1 Introduction

### 1.1 Natural products from marine microorganisms

A large portion of this planet is covered by oceans that harbor a variety of living forms with enormous potential to produce natural compounds. Indeed, marine organisms are reportedly a better source for the production of bioactive compounds than their terrestrial counterparts (Karthikeyan et al., 2022). Life on this planet evolved in a marine environment and hence the marine organisms have witnessed all the evolutionary periods and have acquired the ability to produce diversified compounds with unique structural and functional properties (Jiménez, 2018; Karthikeyan et al., 2022). Approximately 28,500 marine natural products (including polysaccharides, peptides, polyketides, polyphenolic compounds, sterol-like products, and alkaloids, among others) had been identified by the end of 2016 (Blunt et al., 2018). Marine natural products present a broad range of biological activities, including anticancer (Carroll et al., 2023), antibacterial (Mohie et al., 2023), antifungal (El-Hossary et al., 2017), and antiviral effects (Ji et al., 2018). Such a range of activities makes the secondary metabolites promising for the development of novel drug prototypes. Despite the promise of marine microorganisms, the literature has not given them enough attention. As a result, our knowledge of their capacities and bioactive qualities is currently restricted. Unexplored marine worlds characterized by high biodiversity are a resource for discovering new structures with unique characteristics. The long evolution of marine organisms has resulted in organisms with rare genes. The environment in which marine microorganisms must survive includes high pressure (up to 1,100 atmospheres), anaerobic conditions, temperatures below 0°C, high acidity (pH 2.8), and ambient temperatures (above 100°C) in hot springs. It was also necessary to adapt to high salinity, radiation, light, and reduced nutrients (Poli et al., 2017). In such cases, extreme conditions promoted genetic and metabolic diversity in marine microorganisms, leading to specific adaptive mechanisms, especially the synthesis of rare protective compounds. It has now been shown that marine microorganisms can synthesize many unique metabolites with different biological properties, which are expected to be used in the pharmaceutical, cosmetic, and medical industries (Puttaswamygowda et al., 2019). For example, microbial hydrolases work in conditions that result in the deposition or denaturation of proteins produced by mesophilic (terrestrial) microorganisms (Ruginescu et al., 2022). Furthermore, seawater, which is similar to human blood plasma in terms of physiology and chemistry, contains biomolecules with lower toxicity and greater therapeutic effectiveness compared to traditional enzymes. As a result, it is not unexpected that there is a growing number of publications each year on the exploration of marine microorganisms as sources of biologically active substances (Schofield et al., 2015; Gui et al., 2017; Ye et al., 2017; Zhang et al., 2018; Hanif et al., 2019; Sun et al., 2019; Cho et al., 2020; Martyniuk et al., 2020; Pedrosa et al., 2020; Ameen et al., 2021; Chung et al., 2023; Nugraha et al., 2023). About 400 reviews have been published in the last five years with the keyword “marine microorganisms.” Such estimates, however, are “narrowly specific” because marine microorganisms produce antibiotics (Ye et al., 2017; Ameen et al., 2021), antitumor compounds (Gui et al., 2017), enzymes (Sun et al., 2019), polysaccharides (Martyniuk et al., 2020), or those that account for the ability of marine fungi (Schofield et al., 2015; Cho et al., 2020), algae (Nugraha et al., 2023) to synthesize some metabolites. Also, most of these reviews focus on marine microbial biodiversity, the determination of chemical composition, structure of metabolites (Gui et al., 2017; Martyniuk et al., 2020; Pedrosa et al., 2020; Chung et al., 2023; Nugraha et al., 2023) and their biological activity (Gui et al., 2017; Sun et al., 2019; Cho et al., 2020; Ameen et al., 2021; Nugraha et al., 2023). However, the prospects of marine microorganisms for use in biotechnology are not considered.

This review intends to examine and condense recent literature findings on the biotechnological capabilities of marine fungi and bacteria in producing a diverse array of valuable products. These products include surfactants, glyco-and lipopeptides, exopolysaccharides, enzymes, as well as metabolites with various biological activities such as antimicrobial, antitumor, and cytotoxic properties.

#### 1.1.1 Marine bacteria: an untapped resource of novel natural products

The presence of marine microorganisms in the marine habitat is very diverse, including in shallow and deep waters, deep-sea hydrothermal vents, polar regions and in varied coral reefs (Poli et al., 2017). The surface of marine organisms, such as corals, fish, sponges and algae serves as a niche for marine bacteria since these microorganisms frequently have symbiotic or mutualistic relationships with higher organisms (Puttaswamygowda et al., 2019). It is estimated that only 0.01% of marine bacteria have been so far characterized (Ruginescu et al., 2022). Analyses of marine metagenomes indicate a large number of bacteria and archaea phylogenetic groups, that are still unexploited (Martyniuk et al., 2020). True marine bacteria represent a treasure for the discovery of novel bioactive compounds (Pedrosa et al., 2020). However, only 179 novel bioactive compounds were characterized in marine bacteria in 2016 (Blunt et al., 2018). For instance, the marine-derived strain *Micromonospora harpali* produces a group of new spirotetronate analogs (e.g., Microsporanates A (1)–F and tetrocarcins *p* (2), A, B, and AC6H that display antibacterial activity against Gram-positive bacteria (MIC of 0.016 to 8 μg mL^−1^) (Gui et al., 2017). Chromomycins (e.g., chromomycin Ap, (3) isolated from cultures of the marine-derived actinomycete *Streptomyces* sp. MBTI36 showed potent antibacterial activity against Gram-positive bacteria including methicillin-resistant *Staphylococcus aureus* (MRSA) (Cho et al., 2020). The bacterium *Micromonospora* sp. produces thiocoraline (4), a depsipeptide which inhibits DNA polymerase-α, and has been applied for the treatment of cancer in preclinical research (Ameen et al., 2021). Moreover, salinosporamide A (5) isolated from cultures of the marine actinobacterium *Salinispora tropica* (Sun et al., 2019) is in clinical trials for the treatment of multiple myeloma, solid tumors, and lymphomas (Chung et al., 2023; Nugraha et al., 2023).

The bacterium *Streptomyces* sp. P11-238 creates two cyclodepsipeptides known as streptodepsipeptides P11A and P11B. These compounds have been found to slow the growth of various glioma cell lines, with IC_50_ values ranging from 0.1 to 1.4 μM. Streptodepsipeptide P11A specifically has been shown to halt the cell cycle in the G0/G1 phase, induce apoptosis, and reduce the activity of certain tumor metabolic enzymes (HK2, PFKFB3, PKM2, LLS, and FASN) (Ye et al., 2017). (−)-Ecteinascidin 743 (Yondelis), on the other hand, was first discovered in the Caribbean ascidian *Ecteinascidia turbinate* but was later found to be produced by a γ-proteobacterial endosymbiont called *Candidatus endoecteinascidia* frumentensis (Schofield et al., 2015). ET-743 is currently used in medical treatments for advanced soft tissue sarcoma and recurrent platinum-sensitive ovarian cancer when combined with liposomal doxorubicin (Hanif et al., 2019).

These examples show how marine bacteria can manufacture bioactive chemicals that could lead to the creation of brand-new medicinal medicines.

A literature survey between 2017 and 2021 of publications in the top leading scientific journals reporting novel bioactive metabolites from marine bacteria provided the following results. Streptoseomycin (8) has been isolated from cultures of the marine-derived *Streptomyces seoulensis* A01 and displayed very good antibacterial activity against *Helicobacter pylori*, *Lactobacillus acidophilus*, *Bifidobacterium bifidum*, *Eubacterium brachy*, *Propionibacterium acnes*, *Staphylococcus aureus*, *Micrococcus luteus* and *Bacillus subtilis*, with MICs between 2 and 64 μg/mL (Zhang et al., 2018). Pyrroloformamides C (9) and D were isolated from cultures of *Streptomyces* sp. CB02980 and displayed moderate activity against *S. aureus* ATCC 29213, MethicillinResistant *Staphylococcus aureus*, *Escherichia coli*, *Klebsiella pneumoniae* (Zhou et al., 2020). The anthranilate derivatives anthranosides A, B and C (10) have been isolated from cultures of the marine bacterium *Streptomyces* sp. CMN-62. Anthranoside C (10) displayed anti-viral activity with IC_50_ of 171 μM against the influenza A H1N1 virus (ribavirin as positive control, IC_50_ 133 μM) (Che et al., 2018). The alkaloids bulbimidazoles A (11) B and C have been isolated from cultures of the marine bacterium *Microbulbifer* sp. DC3-6 and displayed antibacterial activity against *Kocuria rhizophila* ATCC9341 and against *S. aureus* FDA209P JC-1 (Karim et al., 2020). Only these three articles reporting novel bioactive metabolites from marine bacteria were published in leading chemistry journals between 2017 and 2021, illustrating that the knowledge of marine bacteria secondary metabolism is still very incipient.

Thus, based on the literature review presented in this section, it can be concluded that there are few studies reporting novel bioactive metabolites of marine bacteria. It was found that the properties of secondary metabolites in marine bacteria are poorly understood. However, it is clear that the potential use of marine bacterial metabolites is great, especially for the treatment of malignant tumors, bacterial and viral infections.

#### 1.1.2 Marine fungi: a promising source for bioactive molecules

Obligate and facultative marine fungi are the two categories by which these organisms are conventionally classified. Regarding diversity and ecological significance, this divide is still debatable (Deshmukh et al., 2018). Oversimplified descriptions have left the definition of marine fungus uncertain. It is frequently forgotten that marine mushrooms may adapt to a variety of marine settings, including saltmarshes, hydrothermal zones, and the deep sea (Gladfelter et al., 2019). Nearly all maritime habitats have been shown to include fungi, including sediments (Barone, 2022), the saltwater column (Xu et al., 2018), and combinations with other marine organisms such as sponges, corals, and algae (Zhao et al., 2019; de Sá et al., 2022; Giddings and Newman, 2022; Hafez Ghoran et al., 2023). Both deep and surface seas include marine fungi, which are a reliable source of many healthy substances like antimicrobials, antioxidants, and anticancer chemicals (Deshmukh et al., 2018).

A marine *Aspergillus* sp. fungus produces dehydroxychlorofusarielin B (12), which exhibits antibacterial activity against methicillin-resistant and multidrug-resistant (MDR) *Staphylococcus aureus* (Gladfelter et al., 2019). Linear peptides, simplicilliumtides A (13), E, G, and H were isolated from cultures of *Simplicillium obclavatum* EIODSF 020. Simplicilliumtide A (13) and G showed weak cytotoxicity toward human leukemia HL-60 cell line with IC_50_ values of 64.7 and 100 μM, while simplicilliumtides E and H showed weak cytotoxicity toward the K562 cell line with IC_50_ values of 39.4 and 73.5 μM (Deshmukh et al., 2018). Chaetoxanthone B (14) was isolated from cultures of a marine *Chaetomium* sp. and showed very good antimalarial activity against *Plasmodium falciparum* (IC_50_ = 0.5 μg mL^−1^) and activity against *Trypanosoma cruzi*, the causative agent of Chagas disease (IC_50_ = 1.5 μg mL^−1^) (Barone, 2022). Five new benzophenone derivatives, including a new eremophilane derivative, have been isolated from cultures of marine-derived *Phomopsis lithocarpus* (Xu et al., 2018). These compounds possess a rare naturally occurring aldehyde functionality within this family. One of the new compounds, tenellone H (15), has exhibited cytotoxic activity against HepG-2 and A549 cell lines with IC_50_ values of 16.0 and 17.6 μM, respectively. Another marine-derived fungus, *Aspergillus niger*, produced malformin C (16), which showed strong anti-HIV-1 activity (Hafez Ghoran et al., 2023). Additionally, stachyflin (17), a terpenoid isolated from cultures of marine-derived Stachybotrys, demonstrated modest activity against the influenza A virus (H1N1) with an IC_50_ of 3,910 − 3 μM (Zhao et al., 2019). These examples highlight the potential for research on marine fungal secondary metabolism as a source for developing drug targets since nearly 38% of microbial bioactive metabolites are of fungal origin, but only 5% of the world’s fungal taxa have been described so far (Giddings and Newman, 2022).

The researchers de Sá et al. found that Neosartorya, a type of marine fungi, are closely related to the group *A. fumigatus*. This suggests that the secondary metabolites produced by *Neosartorya* fungi will be similar to those produced by *Aspergillus* species. In fact, they have discovered that there are common features in the secondary metabolites of both *Neosartorya* and *Aspergillus* species, particularly indoles, meroterpenoids, and polyketides (de Sá et al., 2022). They have also identified different types of metabolites within the same species, depending on the environment they are found in. These include indole alkaloids, prenylated indoles, 1,4-benzodiazepen-2,5-dione-containing prenylated indoles, and many others such as peptides, terpenoids, sterols and sterones, polyketides, benzoic acid derivatives, and nucleosides.

Compounds isolated from representatives of the genus *Neosartorya* were tested for biological and pharmacological activity, mainly *in vitro*. Like all other natural products, most of the natural compounds isolated from *Neosartorya* species exhibited anticancer,cytotoxic and antimicrobial activities (de Sá et al., 2022).

## 2 Biologically active marine microorganisms

### 2.1 Methods of isolation of biologically active substances from marine microorganisms

Biologically active substances from marine microorganisms are isolated or purified using preparative column chromatography and high-performance liquid chromatography (HPLC) on direct and reversed phases. The structures of the compounds are determined using state-of-the-art NMR spectroscopy, including ^1^H, ^13^C, COSY, NOESY, HSQC, HMBC, TOCSY, and mass spectrometry (EI, LSI, ESI, MALDI-TOF), including tandem mass spectrometry. Chemical transformations are used to obtain derivatives of biologically active substances from marine microorganisms (Makarieva et al., 2014). Absolute configurations of asymmetric centers are established using various methods. For example, acetylated derivatives of (−)-2-octyl-glycosides of the corresponding monosaccharides are analyzed using gas–liquid chromatography to determine the absolute configuration of the monosaccharides. Relative and absolute configurations of asymmetric side chain centers of new steroids and terpenoids were determined using the modified Mosher method (Makarieva et al., 2014).

### 2.2 Antibacterial and anti-inflammatory effects of marine microorganisms

In the environments where we live, bacteria are extensively dispersed. Infections brought on by pathogenic bacteria have a tremendous impact on human health despite the fact that only a limited number of bacteria may cause them (Packiavathy et al., 2021). Bacterial infections are generally less severe compared to fungal or viral infections (Shafiekhani et al., 2022). However, the issue of antimicrobial resistance is becoming increasingly urgent and surpasses the negative effects of fungal and viral infections (Dadgostar, 2019).

Microbes like bacteria and fungi produce secondary metabolites that are effective against other microbes or particular physiological states in sick organisms. Particularly, bacteria are known for producing a wide variety of structurally distinct bioactive chemicals with powerful biological activity. Consequently, they are regarded as an intriguing source for such compounds (Mulani et al., 2019).

The production of new therapeutic compounds is possible through the bacteria present in seawater, sediments, and marine organisms (Mulani et al., 2019). These marine bacteria utilize nutrients from their host, such as carbon, to protect against environmental pollutants by releasing bioactive chemicals (Schultz et al., 2020).

In a recent study, Contreras-Castro et al. (2020) conducted a recent study on bacteria found in marine sediments in Punta Arena de la Ventana, Mexico. They utilized 16S rRNA gene identification and multilocus sequence analysis to identify 71 isolates as members of the *Salinispora* genus. The researchers then examined the antibacterial properties of various *Salinispora* spp., with 10 demonstrating strong inhibitory activity against ESKAPE infection (Stonik et al., 2020). Out of the tested isolates, 23 were found to be positive for one or more bacterial pathogens. Streptomyces sp. was also found to possess antibacterial activity against drug-resistant bacteria like *E. faecium*, *Staphylococcus aureus*, and *A. baumannii*. A new acidic fluorophore called chlorocaterin, which is a chlorinated catecholic acid, was discovered (Yang et al., 2023). Streptomyces has also shown the ability to produce biologically active compounds like lactic acid and indole-3, which exhibit potent antibacterial activity against *C. albicans* and *Mucor mihei* (Fijan et al., 2022).

*Bacteroidia, Cytophaga, Flavobacteria,* and *Sphingobacteria* represent the four major classes of *Bacteroidetes.* The bacteria are classified as cephalosporin-negative bacteria, bacteriophage bacteria, and non-sporulating bacteria and can survive in both aerobic and anaerobic environments (Li et al., 2019). *Bacteroidetes* bacteria are found not only in marine habitats such as sediments, seawater, and soil, but also in biological habitats such as animal skin and intestines (Huang, 2023).

Cyanobacteria are unique in that they are the sole type of bacteria that generate energy through photosynthesis. Additionally, they are the only group of photosynthetic prokaryotes that are able to generate oxygen and take in CO_2_ (Kariyazono et al., 2022). For a while now, marine cyanobacteria’s secondary metabolites have been recognized for their pharmacological traits, such as their ability to fight off bacteria, viruses, fungi, and more (Perera et al., 2023).

According to studies (Brumley et al., 2018), *Fischerella ambigua*, a cyanobacterium, has an antibacterial effect on *Mycobacterium tuberculosis, Ambigane,* and *Misonitrile*, with values of the minimum limiting concentration ranging from 6.6 to 7.5 μM. New (alkylphenols, anaephenes AC) were isolated from the cyanobacterium *Hormoscilla* sp. They demonstrated moderate inhibition of *S. aureus* growth (Giddings and Newman, 2022). Anaephenes A, B, and C were able to completely inhibit the visible growth of *S. aureus* at concentrations of 22, 6.1, and 22 μg/mL, respectively. When it comes to antiviral drugs isolated from marine bacteria, there are only a few compared to antibacterial and antifungal drugs. Glycolipids are heterosteric compounds that can be found in cyanobacteria and form thylakoid membranes in all prokaryotic and eukaryotic photosynthetic organisms (Brumley et al., 2018). Numerous studies have demonstrated that glycolipids are antiviral agents against the influenza virus (Stincone and Brandelli, 2020). Glycolipids were also found to be effective against HIV. For example, cyanobacteria (*Phormidium tenue* and *Yngbya lagerheimii*) secrete glycolipids containing sulfonic acid, which inhibit HIV-1 reverse transcriptase (Saurav et al., 2017). The cholesterol glycolipids of the cyanobacteria *Oscillatoria laoin and Cytonema* sp. secrete HIV-1 reverse transcriptase at a concentration of 10 μM (Yasir, 2018). Cyanobacterial lipoprotein complexes containing sulfoquinosylparanosillipids inhibit the enzymatic cleavage of HIV-1 virus. At an IC_50_ concentration of 24 nM, esom sulfonide inhibited the HIV-1 DNA polymerase reverse transcriptase pathway in humans. Calcium cyproline is an antiviral that protects against HIV-1, HPV-1, influenza A, and herpes simplex.

Recently, a new antibacterial compound based on oxygenated cycloketones was isolated from rivers containing *B. strocoris*. The isolated compounds inhibited the growth of aquatic bacteria of the genera *Aeromonas* and *Vibrio*. Furthermore, a secondary vaxillin antagonist metabolite has been discovered in the seaweed *B. amyloliquefaciens* MTCC 10456. Wang et al. have published a report on the antibacterial activity of a new thiopeptide antibiotic micrococcin, which was obtained from *B. stratosphericus* in a marine environment. This antibiotic has shown potent activity against Gram-positive bacterial pathogens at concentrations up to 10 μM (Kuo et al., 2019). Marine sponges are known to play a crucial role in benthic communities worldwide, as they can impact both biomass and pelagic processes (Maslin et al., 2021). These sponges are part of a diverse microbial community that includes bacteria, fungi, archaea, and viruses – all of which are valuable sources of natural products. These natural products can be produced by sponges themselves, their microbial symbionts, or through interactions between sponges and symbionts. Consequently, many researchers have focused on studying related bacteria for the purpose of screening and isolating biologically active compounds.

Altuğ et al. demonstrated that methanolic extracts of seaweed-associated bacteria are effective against various bacterial pathogens such as *Staphylococcus aureus, Vibrio vulnificus*, and *Escherichia coli*. Bacterial diseases were studied (Altuğ et al., 2021). About 22% of the biologically active compounds were isolated from marine fungi associated with *Athymycota*. A recent study by Fahmy et al. included the antibacterial compound isolated from *Streptomyces* sp. (Fahmy and Abdel-Tawab, 2021). Cyclopeptides isolated from fungi were applied to *B. cerevisiae* at a concentration of 25 μg/mL. In another study, the new thiopeptide antibiotics YM-266183 and YM-266184 were associated with the marine bacteria *B. cereus* QN03323 and the salmon *Halychondria japonica*. Moreover, both of these thiopeptide antibiotics have demonstrated good inhibitory activity against Gram-positive bacterial infections (Wang et al., 2020).

*Pseudomonas*, a marine bacteria found in association with *Diginea* sp. algae, yielded a novel cyclic tetrapeptide complex, cyclo-[phenylalanyl-prolyl-leucyl-prolyl], which exhibits potent inhibitory effects against *V. anguillarum* and *B. subtilis* (Rungprom et al., 2008). Avila and Angulo-Preckler demonstrated that *P. tunicata*-produced marine bacteria have a competitive edge over their fungi counterparts in surface colonization. Additionally, *P. tunicata*-derived yellow chemical molecule, tamjamine, is believed to possess antibacterial properties (Avila and Angulo-Preckler, 2020) that effectively suppress the growth of staphylococci and bacilli (Tan, 2023).

Another bioactive compound, 2,4-diacetylfloroglucin, was isolated from a new strain of *Pseudomonas*. *Pseudomonas* isolated from *Padina tetrastromatica* algae inhibited the growth of Gram-negative bacterial pathogens such as *Pseudomonas aeruginosa* and *Pseudomonas aeruginosa* at a dose of 300 mg (Arslan et al., 2021). *Pelagiobacter* var*iabilis*, a new marine bacterium isolated from macroalgae, produces a number of chemical molecules of peragomycin from A to C (Srinivasan et al., 2021).

The content of secondary metabolites in marine bacteria exhibiting antibacterial activity is presented in Table 1.

**Table 1:** Content of secondary metabolites of marine bacteria with antibacterial activity.

No.	Marine bacteria	Secondary metabolites	Biological activity	Mechanism of action	References
1	*Bacillus amyloliquefaciens*	Macrolactin V	Antibacterial activity	strong antibacterial activity against *E. coli*, *Bacillus* *subtilis*, and *S. aureus*, with an MIC value of 0.1 μg ml^−1^, and no activity against *Bacillus thuringiensis*	Liu et al. (2019)
2	*Salinispora arenicola*	4-Hydroxy-pyran-2-one and 3-hydroxy-*N*-methyl-2-oxindole derivatives	Antibacterial activity	active against *Enterococcus faecalis* with MIC value of 15.6 μg/mL	Gao et al. (2010)
3	*Bacillus sonorensis* MT93	Sonorensin	Antimicrobial activity	Antimicrobial activity against food-spoiling bacteria, such as *L. monocytogenes* and *S. aureus*	da Silva et al. (2019)
4	*Bacillus cereus*	Thiopeptide antibiotics YM-266183 and YM-266184	Antibacterial activity	antibacterial activities against *staphylococci* and *enterococci* including multiple drug resistant strains, whereas they were inactive against Gram- negative bacteria	Wefky et al. (2018)
5	*Mytilus* *edulis*	Bacicyclin	Antibacterial activity	antibacterial activity against the clinically relevant strains *Enterococcus faecalis* and *Staphylococcus aureus* with minimal inhibitory concentration values of 8 and 12 μM, respectively	Zhang et al. (2023)
6	*Streptomyces sampsonii*	Julichromes	Antimicrobial activity	against Gram-positive pathogens, including *Micrococcus luteus* and *Bacillus subtilis*, with MIC values ranging from 2 to 64 μg/mL	Sannino et al. (2017)
7	*Pseudomonas putida*	9, 10 dihydrophenanthrene-2-carboxylic acid	Antifungal activity	active against fungal strains obtained from clinical samples whereas strong activity was noted against *Candida albicans* with a MIC value of 20 μg/mL	Uzair et al. (2018)
8	*Diginea* sp.*, Pseudomonas*	Cyclo-[phenylalanyl-prolyl-leucyl-leucyl-prolyl] (A), cyclo-[isoleucyl-prolyl-leucyl-alanyl](B)	Antibiotic activity	antimicrobial activity when tested against *Staphylococcus aureus*, *Micrococcus luteus*, *Bacillus subtilis*, *Escherichia coli* or *Vibrio anguillarum*	Islam et al. (2022)
9	*Hormoscilla* sp.	Anaephenes	Antibacterial and Antifungal Activities	Anaephenes A&C were elucidated by spectroscopic methods, and compounds assayed for growth inhibitory activity against prokaryotic and eukaryotic cell lines. Anaephene B displayed moderate activity against *Bacillus cereus* and *Staphylococcus aureus* with MIC values of 6.1 μg/mL	Ng et al. (2020)
10	*P. tenue* and *Y. lagerheimii*	Sulfonic acid-containing glycolipids	Anti-HTV-1	active against HIV-1 in cultured human lymphoblastoid CEM, MT-2, LDV-7, and C3-44 cell lines	Ng et al. (2020)
11	*Nostoc ellipsosporum*	Cyanovirin-N	anti-HIV microbicide	Interfering with the interaction of the HIV gp120 with the CD4 T-cell receptor	Ng et al. (2020)

It is known that various mediators, such as prostaglandins, leukotrienes and kinins, platelet activation factors are involved in the development of inflammation. The study (Srilekha et al., 2017) presents the results of carrageenan injection from the marine bacterium *Brevibacterium* sp. in inflammation, indicating that the inflammatory mediator serotonin is released in phase 1, causing inflammation. This first phase occurs within the first hour after carrageenan injection. Prostaglandins, inflammatory mediators, are released in phase 2 and begin to exert their inflammatory effects around the third hour of the inflammatory process.

Oxidative stress plays an important role in endothelial dysfunction (Miksch et al., 2021), lung disease, gastrointestinal dysfunction, and atherosclerosis, all of which are associated with inflammatory responses (Ribeiro et al., 2023). The crude extract of pigment was used as an anti-inflammatory agent to induce paw edema in male Wistar rats. A significant reduction in edema and inflammation was noted. Marine bacteria are considered very valuable because they produce various antibiotics and other therapeutically useful compounds with diverse biological activities (Anguita-Maeso et al., 2020).

Chemical extracts of marine Actinobacteria isolates were screened for their antimicrobial and anti-inflammatory activities as described in (Ribeiro et al., 2023). Extracts of two *Streptomyces* strains showed activity against *Candida albicans*. In addition, 15 extracts (derived from marine microorganisms *Brachybacterium, Brevibacterium, Microbacterium, Microbacterium, Rhodococcus,* and *Streptomyces*) demonstrated anti-inflammatory potential in the RAW 264.4 cell model assay without concomitant cytotoxic response.

Elbandy discovered that neuroinflammation is closely linked to the development and progression of various neurodegenerative conditions such as Alzheimer’s disease, Huntington’s disease, and Parkinson’s disease (Elbandy, 2023). The activation of astrocytes and microglia serves as the brain’s defense mechanism against damaged tissue and harmful pathogens, but their chronic activation can lead to neuroinflammation, which in turn worsens or triggers neurodegeneration. Currently available therapeutic drugs only provide temporary relief for these disorders, and there are no treatments available that can slow down or stop the progression of neurodegeneration. Therefore, natural compounds that may have a protective effect against these disorders hold therapeutic potential. Marine sources have already yielded numerous chemical compounds such as bioactive peptides, fatty acids, pigments, alkaloids, and polysaccharides which possess anti-inflammatory properties and can be effective in treating and preventing neuroinflammatory diseases. The anti-inflammatory components found in marine compounds are utilized as functional food ingredients for preventing and treating neurological disorders (Elbandy, 2023).

### 2.3 Antibiofilm activity of marine microorganisms

A biofilm is an aggregation of microorganisms attached to a surface (O’Toole et al., 2000). It consists of microbial cells encapsulated in an extracellular polymeric matrix containing various biopolymers such as proteins, nucleic acids, lipids and other substances (Flemming and Wingender, 2010). Biofilms consist of one or more species of microorganisms colonizing biological surfaces (Hall-Stoodley et al., 2004). The complex biofilm structure protects microorganisms within the biofilm and provides the spatial proximity and internal communication necessary for growth and development (Dang and Lovell, 2016). This film can protect microbial cells from various external influences, such as antimicrobial therapy, toxins, antiprotozoal therapy, and host immune defense. For example, biological preparations reduce the sensitivity of microorganisms to antimicrobial drugs by 10–1,000 times. In addition, multispecies organisms are less susceptible to antimicrobial therapy than single-species organisms due to complex host-organism interactions (Hengzhuang et al., 2011).

In the marine environment, microorganisms rapidly colonize living and abiotic surfaces and subsequently form a biota consisting of bacteria, diatoms, fungi, unicellular algae and protozoa (Qian et al., 2007), which is a serious problem for humans. Marine biomass plays an important role in the biodegradation of the marine environment. Biomass plays an important role in the habitat selection and distribution of many surface-dwelling marine species. For example, invertebrate larvae can differentiate between biomes composed of different microbial community structures that may or may not form colonies (Dobretsov and Qian, 2006). Various substances are broken down by microbial metabolites such as hydrogen sulfide, various acids, and ammonia. Every year, biofouling and biodegradation cause significant economic losses worldwide in industries such as heat transfer, oil and gas processing, storage, and transportation, and drinking water and wastewater treatment (De Carvalho, 2018; Plaza and Achal, 2020). Furthermore, the persistence and spread of harmful or pathogenic microorganisms and their genetic material in marine biota poses a serious threat to humans (Hengzhuang et al., 2011).

**Figure 1:**
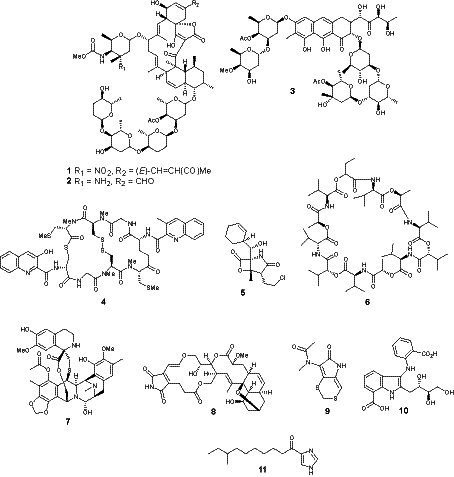
Bioactive metabolites isolated from marine bacteria.

Given the increasing economic losses and potential risks of marine biofilm formation, effective and efficient control mechanisms are needed. Antibiotic/antioxidant coatings are now widely used and provide a simple method of controlling the marine environment. However, chemical inhibitors such as amines, amides, organotin compounds and copper oxides used in traditional adhesives are toxic and harmful, have no favorable environmental impact and are prone to biological oxidation (Figure 1). Special attention is paid to the development of effective and safe adhesives using natural products. Natural compounds with antimicrobial activity produced by the metabolic mechanism of microorganisms can replace traditional chemical and biological agents and have environmentally friendly properties such as low toxicity and biodegradability. However, insufficient funding and complexity limit the development of naturally synthesizable composites (Ohno et al., 1978).

**Figure 2:**
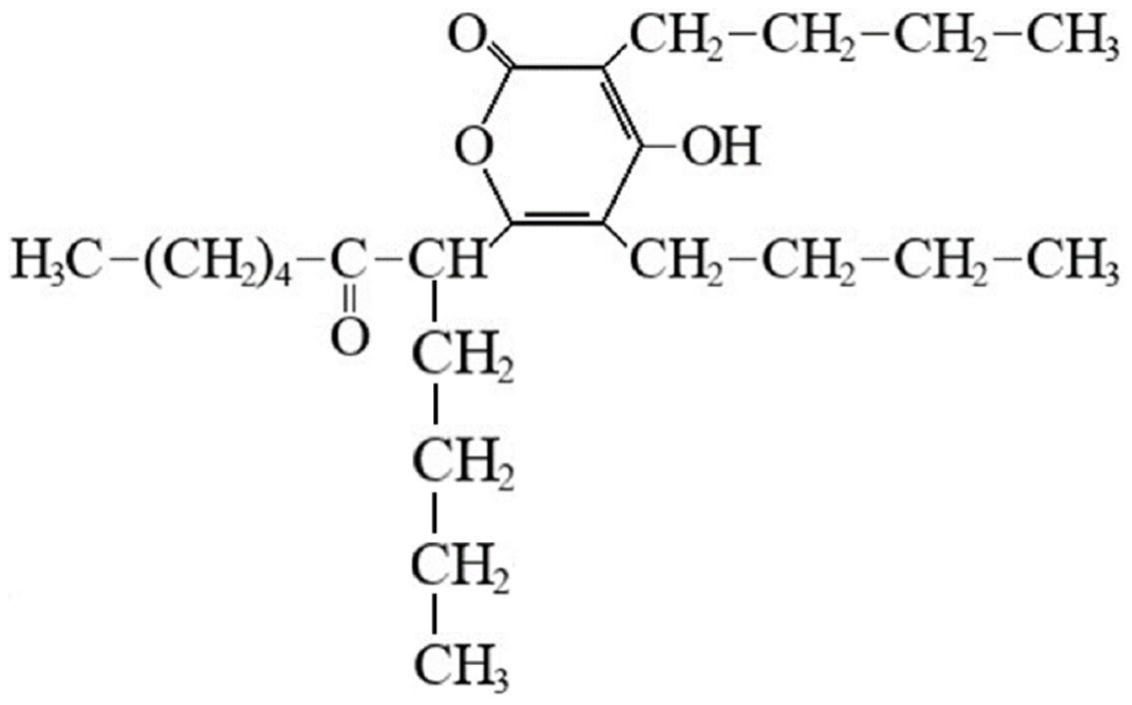
Structural formula of elasnin.

Bacteria, as major colonizers in marine biota, are important determinants of the structure and function of mature biomass (De Carvalho, 2018). Thus, marine biofilm bacteria may be an excellent target for the discovery of compounds that inhibit biofilm formation. Compounds that inhibit biofilm formation are being sought using bacteria isolated from marine biofilms, leading to the discovery of potent activity of elasnin against biofilms. The structural formula of elasnin is shown in Figure 2.

In a screening program of antibiofilm compounds against marine biofilms, a potent biofilm inhibitory activity of elasnin was found (Hengzhuang et al., 2011). Elasnin effectively inhibited biofilm formation of seven bacterial strains isolated from marine biofilms. With high performance, elastin-based coatings were fabricated in a simple and cost-effective manner that showed excellent results in suppressing the formation of multispecies biofilms and attachment of large biofouling organisms in marine environments. 16S-amplicon and anti-larvae assays showed that elasnin could prevent biofouling through indirect effects of altered microbial biofilm composition and direct inhibitory effects on larval settlement with low toxic effects. These results indicate the potential application of elasnin for biofilm and biofouling control in marine environments (Hengzhuang et al., 2011).

Ten strains isolated from marine biofilms (*Vibrio alginolyticus* B1, *Erythrobacter* sp.HKB8, *Rugeria* B32, *Staphylococcus aureus* B04, *Staphylococcus hominis* N32, *Staphylococcus arlettae* OM, *Microbacterium asteromaticum* N22, *Idiomarina sediminum* N28, *Pseudoalteromonas* L001, and *Escherichia coli* N57) were used as targets in the analysis of minimum biofilm inhibitory concentrations (MIC). MBIC is the lowest concentration of a compound that specifically reduces the number of attached cells, whereas MIC is the lowest concentration required to inhibit significant planktonic cell growth. Seven of the ten strains tested formed biofilms successfully during the experiment, while three strains (*V. alginolyticus* B1, *Erythrobacter* sp. HKB8, and *Rugeria* B32) failed to form biofilms under the experimental conditions. For the seven biofilm-forming strains, the biofilms of the four Gram-positive bands were sensitive to arginine levels: MBIC 90 was between 2.5 and 5 mg/mL, and MBIC 50 was between 1.25 and 5 mg/mL. Both MBIC 90 and MBIC 50 g were sensitive to enzyme action in a concentration-dependent manner. Enzyme concentrations ranged from 5 to 10 mg/mL and 1.25 to 10 mg/mL, respectively. The BICs of nine strains were determined, except for *M. asteraromaticum* N22, which failed to grow under the experimental conditions. *Elasnin* inhibited the growth of planktonic cells of *S. aureus* B04 and *I. sediminum* N28 with BICs between 5 and 10 mg/mL, whereas the BIC of elasnin exceeded 10 mg/mL for the other species. Overall, elasnin inhibits biofilm formation more effectively than it does antibacterial activity.

**Figure 3:**
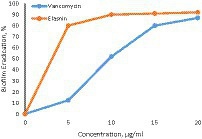
Dependence of biofilm eradication degree on elastin concentration (vancomycin – control).

The dependence of the degree of biofilm eradication on elastin concentration is presented in Figure 3.

As a result, a highly effective elasnin-based coating was developed and its activity against a wide range of natural biological agents was evaluated in clinical trials (Long et al., 2021).

Elasnin was first discovered in 1978 as a novel elastase inhibitor with low toxicity to rats and high selectivity for human granulomatous elastase (Nsanzabana, 2019), but its antibacterial and antibiotic activity was not detected at that time. According to the experimental results, elasnin not only inhibited the formation of single and multi-organism biofilms, but also inhibited macroorganism colonization. 16S-amplicon analysis suggests that the altered biome may not promote microbial colonization, so elasnin-based biofilms may reduce coat biodiversity, indirectly limiting biocolonization. In addition, antibody assays showed that elasnin inhibited larval colonization with low toxicity. Considering the effective concentration of elasnin and its mobility in the marine environment, the toxic effects of elasnin should be ignored. Elasnin shows great potential for biocide and biological control applications in the marine environment due to its low toxicity, high efficacy and high productivity (Hengzhuang et al., 2011).

### 2.4 Antimalarial action of marine microorganisms

More than 40% of the world’s population now lives in areas where malaria is common. There were 219 million clinical cases of malaria in 2017, and 435,000 people died from the disease (Lackner et al., 2017). Existing antimalarials quickly become ineffective due to the rapid emergence of resistant bacteria. Artemisinin therapy is recommended for the treatment of malaria even in malaria-endemic countries, but widespread resistance has been shown to be alarming in Southeast Asia (Newman and Cragg, 2020). Given the alarming situation in malaria treatment, there is an urgent need for new chemotherapeutic agents for next-generation antimicrobial therapy.

Throughout history, natural products have played a crucial role in uncovering new potential for pharmaceuticals. Between 1981 and 2014, a total of 1,562 fresh drugs were created, with 59.5% being derived from natural raw materials (WHO, 2020). Natural products are particularly valuable as sources of anti-cancer and antimicrobial agents, such as quinine-based antimalarials. Artemisinin, which was initially used in phytotherapy and is derived from pepper, has recently emerged as a leading treatment for drug-resistant malaria. Even though most approved natural products and naturally occurring chemicals come from terrestrial habitats, the marine environment remains largely uncharted. This presents an opportunity to discover new therapeutic compounds from a variety of marine organisms. Invertebrates and algae have traditionally been the primary targets for discovering new therapeutic medications, but marine biotechnology is now focusing on marine microbes (Domínguez-Oliva et al., 2023).

Many compounds originally isolated from marine invertebrates are thought to come from marine bacteria (McCarthy et al., 2019). Marine surfaces usually contain 10 ^5^ microbial cells per mL (Nielsen et al., 2017). The so-called microbial decoctions of some algae contain a large number of related microorganisms with a density of 10^8^ to 10^10^/g, which amounts to 20 to 30% of the mollusc biomass. There is growing evidence indicating that these communities of microorganisms play a crucial role in the metabolism of molluscs, including the production of natural substances (Nicoletti et al., 2018). Manzamin A, an antiviral substance produced by actinomycetes associated with fungi, is one of the earliest examples of mollusc metabolites that have been demonstrated to be produced by cultivated microorganisms (Ruiz-Gil et al., 2020). Marine microorganisms have been found to produce various antibacterial compounds. For instance, *Pseudomonas* (Masschelein et al., 2017) associated with sponges produces 2-undecyl-4-quinolone while *Calothrix* (Dyshlovoy and Honecker, 2018), a type of cyanobacterium, produces calotrixins A and B. Despite the potential of the marine microbiome for discovering new chemotypes, it has not been fully utilized for discovering new antimalarials.

A study (Bunbamrung et al., 2020) mentions that the phylogenetic relationships of 2,290 heterotrophic marine bacterial isolates were determined by analyzing restriction fragment length polymorphism and 16SU rRNA gene sequences (Meyer et al., 2020). The resulting similarity was <97%. Common fungi isolated from the sea, such as *Penicillium* and *Aspergillus*, have been found to produce new metabolites (Xu et al., 2020).

Marine bacteria that have antimalarial activity come from many taxa. There are five known isolates of *Penicillium* fungi (Tajuddeen and Van Heerden, 2019). There is a wealth of information available on terrestrial penicillins and other naturally occurring fungi-derived products (Trial.gov, C, 2023). Recently, however, marine isolates of these fungi have become a source of new natural antimalarial products (Álvarez-Bardón et al., 2020). The same applies to common mangrove endophytes, which have been found to produce a wide range of natural antimalarial products, including xantoquinidin-like tritilachium compounds isolated from marine fungi (Bank, D, 2019).

Six active marine actinomycetes (Gram-positive bacteria) usually produce biologically active antimalarial products (Miksch et al., 2021). In screening studies, six isolates of marine Gram-negative bacteria were found to have antimalarial activity (da Silva et al., 2019). Recently, marine gram-negative bacteria have become recognized as a valuable source for antimalarial products, including polyketides, non-ribosomal peptides, and polyketide-neribosomal hybrid peptides (Wefky et al., 2018). Of the three active isolates discovered, all belonged to the genus Marinobacter and demonstrated the ability to produce the siderophore petrobactin (Dyshlovoy and Honecker, 2018).

The oil-degrading bacterium Alcanivorax is known to produce the active ingredients and α-pyrone alkaniborone (ClinicalTrials.gov, 2023a), but the biological activity of these molecules has not been reported. *Endozoiconado* spp. can be associated with marine fungi, sponges, and octocorals and can dominate microbial populations. Although their secondary metabolites have not been studied, one article describes the antimalarial activity of endozoicomonad extracts (Martyniuk et al., 2020).

These results support the hypothesis that marine microbes can produce new antimalarial chemicals. Based on literature review (Bank, D, 2019; ClinicalTrials.gov, 2023a; DrugBank Online, 2023a), the following marine organisms were found to have significant antimalarial activity: *Streptomyces* sp., *Nocardiopsis* sp., *Micromonospora* sp., *Unidentified actinomycete*, *Penicillium* sp., *Endozoicomonas numazuensis*, *Alcanivorax* sp., *Marinobacter* sp., *Talaromyces rotundus*, and *Tritirachium* sp. Taxonomic diversity is achieved among individuals with high levels of activity, and it is expected to develop into chemical diversity of antimalarials as research progresses (ClinicalTrials.gov, 2023a). Table 2 presents the activity of various marine microorganisms against malaria.

**Table 2:** Antimalarial activity of marine microorganisms.

No.	Microorganism	Activity period, days	Antimalarial cytotoxicity	References
1	*Streptomyces* sp.	14	29.1	ClinicalTrials.gov (2023a)
2	*Nocardiopsis* sp.	21	9.1	ClinicalTrials.gov (2023a)
3	*Micromonospora* sp.	14	9.3	ClinicalTrials.gov (2023a)
4	*Unidentified actinomycete*	21	10,2	ClinicalTrials.gov (2023a)
5	*Penicillium* sp.	22	>50	ClinicalTrials.gov (2023a)
6	*E. numazuensis*	7	>50	DrugBank Online (2023b)
7	*Alcanivorax* sp.	7	>50	Newman and Cragg (2004)
8	*Marinobacter* sp.	24	>50	Newman and Cragg (2004)
9	*T. rotundus*	21	>50	ClinicalTrials.gov (2023a)
10	*Tritirachium* sp.	7	>50	ClinicalTrials.gov (2023a)

### 2.5 Anticarcinogenic effect of marine microorganisms

It has been found that bioactive molecules derived from nature have the ability to eliminate cancer cells by targeting their macromolecules in oncogenic signaling pathways (Bank, D, 2019). Numerous metabolites obtained from the ocean have demonstrated the ability to impede the growth of human tumor cells in both laboratory and animal models (specifically rats), as well as in clinical trials (ClinicalTrials.gov, 2023a; DrugBank Online, 2023a). By utilizing advanced technology and natural marine products, researchers have come across a new generation of anticarcinogenic agents that are currently undergoing clinical testing (DrugBank Online, 2023b). Marine microbes possess immense potential for the discovery of new substances that can be advantageous for cancer prevention and treatment. The diversity of bacteria found in natural marine products is increasingly attracting attention for its ability to overcome challenges in drug discovery (Newman and Cragg, 2004). In general, natural products serve as a significant source of compounds that can effectively treat various forms of cancer and present opportunities for exploring novel mechanisms of action (Khotimchenko, 2010).

**Figure 4:**
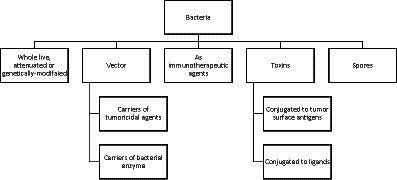
Schematic overview of the role of marine bacteria in cancer therapy.

Live non-pathogenic marine bacterial species can selectively proliferate in tumors and inhibit their growth. More than 70 compounds belonging to different structural classes of marine microorganisms, such as polyketides, indolocarbozoles, isoprenoids, macrolides, non-ribosomal proteins, etc., have been proven to have antitumor effects on various cell lines (Zong et al., 2023). Due to their selectivity towards tumor tissues, these marine bacteria and their spores (Zong et al., 2023) also serve as ideal vectors for the delivery of therapeutic proteins to tumors. Marine bacterial toxins have also emerged as a promising strategy for cancer treatment (Rommasi, 2022). The most potential and promising strategy is marine bacteria-based gene-directed enzymatic prodrug therapy. This method has been shown to be effective in the treatment of cancer *in vivo* (Jha and Zi-rong, 2004). Marine microorganisms produce unusual macrolactins that inhibit proliferation of melanoma B16-F10 cells in rodents, suppress replication of mammalian herpes simplex viruses, and protect T lymphocytes from human immunodeficiency virus (HIV) (DrugBank Online, 2023c). A schematic overview of the role of marine bacteria in cancer therapy is presented in Figure 4.

Over the course of the last half-century, data has indicated that a variety of natural products derived from marine plants and microbes (as listed in Table 3) have promising potential for preventing and treating cancer. This includes medications such as cytarabine, eribulin mesylate, brentuximab vedotin, and trabectedin, which have been utilized to treat leukemia, breast cancer, soft tissue sarcoma, and cervical cancer (ClinicalTrials.gov, 2023b). To accelerate the development of new anti-tumor drugs that are highly effective with minimal side effects from marine resources, a comprehensive integrated approach has been proposed. The marine organisms examined in this review (*Streptomyces* sp.*, Aplidium albicans, Nostoc* sp.*, Dolabella auricularia, Ecteinascidia turbinate, Halichondria okadai, Bryopsis* sp.*, Elysia rufescens, Sponges, Mollusc*) had high anticancer activity (Table 3).

**Table 3:** Potential anti-cancer effects of marine sources.

No.	Marine source	Cancer type	References
1	*Streptomyces* sp.	Pediatric cancer, Wilms tumor	Ng et al. (2020)
2	*A. albicans*	Pancreatic, stomach, bladder, and prostate cancer	ClinicalTrials.gov (2023b)
3	*Nostoc* sp.	Leukemia	ClinicalTrials.gov (2023c)
4	*D. auricularia*	Pancreatic cancer	ClinicalTrials.gov (2023c)
5	*E. turbinate*	Sarcomas and ovarian cancer	ClinicalTrials.gov (2023c)
6	*H. okadai*	Advanced breast tumors	Khalifa et al. (2019)
7	*Bryopsis* sp.	Prostate cancer	Atanasov et al. (2021)
8	*E. rufescens*	Breast cancer, colon cancer	Naeem et al. (2022)
9	Sponges	Lung and prostate cancer	Bayer et al. (2013)
10	Molluscs	Pancreatic cancer	Rua et al. (2014)

A research study has reported the identification of a vast number of microorganisms, approximately 3.7 × 10^30^ derived from marine organisms (Pike et al., 2013). Although most of these bacteria cannot be grown in laboratory conditions, they have the ability to produce numerous natural compounds with potential medical applications (Nobili et al., 2009). The impressive chemical and pharmacological capabilities observed in marine organisms can be attributed to their adaptation to extreme conditions, which prompts them to synthesize secondary metabolites that are resistant to heat, salt, stress, and predators. In the past century, various marine organisms including bacteria, actinomycetes, cyanobacteria, fungi, microbes, macromycetes (Shahidi and Santhiravel, 2022), invertebrates, molluscs, soft corals, sea sponges, sea urchins, holojamberts, shellfish, and other sea creatures have been discovered and extensively investigated for their anticancer properties (Bhanot et al., 2011). Furthermore, advancements in marine chemistry have facilitated the utilization of metabolomics and other novel tools to gain new insights into aquatic products (Obaid et al., 2023).

Microbes are responsible for the production of more than 22,000 secondary metabolites, with actinomycetes accounting for 70%, fungi for 20%, *Bacillus* spp. for 7%, and other bacteria for 1–2% (Rocha-Martin et al., 2014). However, only a small portion (10%) of biologically active natural products can be attributed to microbial sources. Several anticancer drugs, including briostatin 1, ET-743, and dolastatin 10, have undergone successful clinical trials. Briostatin 1 recently completed phase II clinical trials and showed efficacy in treating melanoma, Hodgkin’s lymphoma, kidney cancer, and colorectal cancer (Boopathy and Kathiresan, 2010). Its mechanism of action involves promoting the proliferation of normal osteoblasts (White et al., 2014). ET-743 is a tetrahydroisoquinolone alkaloid derived from erythropoietin that has passed phase I clinical trials due to its selective targeting of guanine residues in the small DNA groove. *Dolabella aureus*, a mollusc peptide, has progressed to phase II clinical trials by inhibiting microbial aggregation and inducing cell cycle metaphase arrest (White et al., 2014).

Cyanobacteria, also known as blue-green algae, offer a diverse range of more than 400 newly discovered metabolites. Among these metabolites, distinct bioactive peptides and polyketides (Bayona et al., 2022) have demonstrated the ability to induce apoptosis in cancer cells and positively impact cellular signal transduction through the protein kinase C family (Ngamcharungchit et al., 2023). In experiments involving 41 cyanobacterial strains, approximately half exhibited the capability to induce cell death in cancer cells (Ngamcharungchit et al., 2023). Dolastin 10 and curazine A, derived from cyanobacteria and possessing antibacterial properties, are currently being clinically evaluated for their potential in cancer therapy and have served as foundational structures for the development of several synthetic analogues and derivatives (Gao et al., 2021). Calothrix A and B are five metabolites isolated from *Cyanobacterium calothrix* that exhibit potent anti-tumor activity against human HeLa cancer cells in virological studies, with IC_50_ values of 40 and 350 nM, respectively. Additionally, Uritiaciclamide synthesized by the cyanobacteria *Prochromon* spp. and pateramide synthesized by *Lissoclinum patella* have demonstrated significant cytotoxic activity against human nasopharyngeal carcinoma cell lines, with IC_50_ values of 17 and 3,000 ng/mL, respectively, (Premarathna et al., 2024).

Borofins, which are metabolites containing boron, have been obtained from the marine cyanobacteria *Nostoc linckia* and *Nostoc spongiaeforme* var. tenue (Encarnação et al., 2015); these compounds have shown strong cytotoxic effects against human epidermal carcinoma (LOVO) and human colorectal adenocarcinoma (KB) cell lines. The septic toxicity of *Nostoc* sp. has also been observed in human tumor cells (GSV 224) and tumor cells, with an IC_50_ value of 0.005 for KB and LOVO cell lines. The *Leptolyngbya* strain exhibits significant cytotoxicity (LC50 < 23 nM) against NCIH460 cells and rat lung neuronal cells. These cells have the ability to regulate enzymatic kinases and inhibit the growth of human fibroblasts and endothelial cells. Curacin A, extracted from organic materials found in the *Lyngbya majuscule* collection in Curaçao, is a notable antiviral agent with strong potency. It can prevent tubulin polymerization and selectively inhibit Burkitt leukemic lymphocytes (Robles-Bañuelos et al., 2022). Apratocin is another cyanobacterial compound that shows inhibition of various cancer cells at nanomolar doses.

Autophagic activity against chronic myelogenous leukocytes was observed in various strains of cyanobacteria, while benign cells such as hepatocytes and cardiomyocytes remained unaffected (Tan, 2007). Current research indicates that benthic cyanobacteria cultivated in a temperate marine environment hold great promise as a previously untapped source of novel anti-leukemia drugs (Khotimchenko, 2010). Nonetheless, certain compounds isolated from marine sources, including calcroxin A, B, euletiaclamide, betylamide, borofin, and largasol, have not yet undergone clinical trials and exhibit distinct modes of action. Consequently, further investigation is required to determine their potential biological activity and potential for clinical application.

Biologically active compounds derived from marine *Pseudomonas* are diverse and include pyrroles, pseudoanes, pyrrolidenediones, floroglucines, phenazines, benzaldehydes, quinines, quinolones, phenanthenes, phthalates, anhydrides, moiramides, chaffrin, and bouchrin. Some of the biologically active compounds in these antimicrobials are dibutyl phthalate and di (2-ethylhexyl) phthalate, which were reported to be cathepsin B inhibitors (Zong et al., 2023). Discodermold, briostatin, sarcoidin, and eletrobin are among the antibiotics produced mainly by marine bacteria (Naeem et al., 2022).

## 3 Conclusion

Based on the generalization of literature sources, it was found that marine microorganisms are a more valuable source of biologically active secondary metabolites than terrestrial ones. This is attributed to the fact that marine organisms have therefore survived all periods of evolution and have acquired the ability to produce a variety of compounds with unique structural and functional properties (Barzkar et al., 2017, 2018, 2019, 2021a,b,c, 2022a,b, 2023a,b; Jahromi and Barzkar, 2018a,b; Barzkar, 2020; Barzkar and Sohail, 2020).

However, only 179 new bioactive compounds have been characterized in marine bacteria. Therefore, the relevance of studying the types and properties of secondary metabolites in marine microorganisms is beyond doubt. The scientific novelty of the study was that for the first time, data on new resources for obtaining biologically active natural productsmetabolites of marine bacteria and fungi - were generalized. The review presents information on types of natural products derived from marine microorganisms (bacteria and fungi), summarizes data on the antibacterial, antimalarial, anticarcinogenic, and anti-inflammatory effects of marine microorganisms. Properties and mechanisms of antibacterial action of the following marine microorganisms have been studied: *Pseudomonas* sp., *M. producens, B. amyloliquefaciens, S. arenicola, B. sonorensis* MT93, *B. cereus, M. edulis, S. sampsonii, P. putida, Diginea* sp., *Pseudomonas, Hormoscilla* sp., *P. tenue* and *Y. Lagerheimii,* and *N. ellipsosporum*. Therefore, there is a great need for scientific and applied research on the bioactive secondary metabolites of marine microorganisms from the viewpoint of human and animal health.

## Author contributions

NB: Conceptualization, Writing – original draft, Writing – review & editing. SS: Writing – review & editing. OB: Writing – original draft, Writing – review & editing.

## References

[ref1] Altuğ G. Çiftçi Türetken P. S. Kalkan S. Topaloğlu B. (2021). The distribution and antibacterial activity of marine sponge-associated bacteria in the Aegean Sea and the sea of Marmara Turkey. Curr. Microbiol. 78, 2275–2290. doi: 10.1007/s00284-021-02489-7, PMID: 33929605

[ref2] Álvarez-Bardón M. Pérez-Pertejo Y. Ordóñez C. Sepúlveda-Crespo D. Carballeira N. M. Tekwani B. L.. (2020). Screening marine natural products for new drug leads against Trypanosomatids and malaria. Mar. Drugs 18:187. doi: 10.3390/md18040187, PMID: 32244488 PMC7230869

[ref3] Ameen F. AlNadhari S. Al-Homaidan A. A. (2021). Marine microorganisms as an untapped source of bioactive compounds. Saudi J. Biol. Sci. 28, 224–231. doi: 10.1016/j.sjbs.2020.09.052, PMID: 33424301 PMC7783642

[ref4] Anguita-Maeso M. Olivares-García C. Haro C. Imperial J. Navas-Cortés J. A. Landa B. B. (2020). Culture-dependent and culture-independent characterization of the olive xylem microbiota: effect of sap extraction methods. Front. Plant Sci. 10:1708. doi: 10.3389/fpls.2019.01708, PMID: 32038682 PMC6988092

[ref5] Arslan E. Çobanoğlu Ş. Yazici A. (2021). Antimicrobial activity of pigments extracted from Auxenochlorella protothecoides SC3 against *Pseudomonas aeruginosa* Tr. J. Nature Sci. 10, 163–167. doi: 10.46810/tdfd.930388

[ref6] Atanasov A. G. Zotchev S. B. Dirsch V. M. (2021). Natural products in drug discovery: advances and opportunities. Nat. Rev. Drug Discov. 20, 200–216. doi: 10.1038/s41573-020-00114-z, PMID: 33510482 PMC7841765

[ref7] Avila C. Angulo-Preckler C. (2020). Bioactive compounds from marine Heterobranchs. Mar. Drugs 18:657. doi: 10.3390/md18120657 33371188 PMC7767343

[ref8] Bank, D (2019). D. B. A. o. h. w. d. c. D. a. o. A., 27 May 2023.

[ref9] Barone G. (2022). Local environmental conditions promote high turnover diversity of benthic Deep-Sea Fungi in the Ross Sea (Antarctica). J. Fungi 8:65 8, 65. doi: 10.3390/jof8010065 PMC8781733 35050005

[ref10] Barzkar N. (2020). Marine microbial alkaline protease: an efficient and essential tool for various industrial applications. Int. J. Biol. Macromol. 161, 1216–1229. doi: 10.1016/j.ijbiomac.2020.06.072, PMID: 32534091

[ref11] Barzkar N. Attaran F. G. Taheri A. (2017). Proximate composition and mineral contents in the body wall of two species of sea cucumber from Oman Sea. Environ. Sci. Pollut. Res. 24, 18907–18911. doi: 10.1007/s11356-017-9379-5, PMID: 28656569

[ref12] Barzkar N. Homaei A. Hemmati R. Patel S. (2018). Thermostable marine microbial proteases for industrial applications: scopes and risks. Extremophiles 22, 335–346. doi: 10.1007/s00792-018-1009-8 29442247

[ref13] Barzkar N. Jahromi S. T. Poorsaheli H. B. Vianello F. (2019). Metabolites from marine microorganisms, micro, and macroalgae: immense scope for pharmacology. Mar. Drugs 17:464. doi: 10.3390/md17080464, PMID: 31398953 PMC6723029

[ref14] Barzkar N. Jahromi S. T. Vianello F. (2022a). Marine microbial fibrinolytic enzymes: an overview of source, production, biochemical properties and thrombolytic activity. Mar. Drugs 20:46. doi: 10.3390/md20010046, PMID: 35049901 PMC8779250

[ref15] Barzkar N. Khan Z. Jahromi S. T. Pourmozaffar S. Gozari M. Nahavandi R. (2021a). A critical review on marine serine protease and its inhibitors: a new wave of drugs? Int. J. Biol. Macromol. 170, 674–687. doi: 10.1016/j.ijbiomac.2020.12.134, PMID: 33387547

[ref16] Barzkar N. Rungsardthong V. Tamadoni Jahromi S. Laraib Q. das R. Babich O.. (2023a). A recent update on fucoidonase: source, isolation methods and its enzymatic activity. Front. Mar. Sci. 10:1129982. doi: 10.3389/fmars.2023.1129982

[ref9001] Barzkar N. Sukhikh S. Babich O Venmathi Maran B. A. Tamadoni Jahromi S. (2023b). Marine collagen: purification, properties and application Front. Mar. Sci 10, 1245077. doi: 10.3389/fmars.2023.1245077, PMID: 33387547

[ref17] Barzkar N. Sheng R. Sohail M. Jahromi S. T. Babich O. Sukhikh S.. (2022b). Alginate lyases from marine bacteria: an enzyme ocean for sustainable future. Molecules 27:3375. doi: 10.3390/molecules27113375, PMID: 35684316 PMC9181867

[ref18] Barzkar N. Sohail M. (2020). An overview on marine cellulolytic enzymes and their potential applications. Appl. Microbiol. Biotechnol. 104, 6873–6892. doi: 10.1007/s00253-020-10692-y, PMID: 32556412

[ref19] Barzkar N. Sohail M. Tamadoni J. S. Gozari M. Poormozaffar S. Nahavandi R.. (2021b). Marine bacterial esterases: emerging biocatalysts for industrial applications. Appl. Biochem. Biotechnol. 193, 1187–1214. doi: 10.1007/s12010-020-03483-8, PMID: 33411134

[ref20] Barzkar N. Sohail M. Tamadoni J. S. Nahavandi R. Khodadadi M. (2021c). Marine microbial l-glutaminase: from pharmaceutical to food industry. Appl. Microbiol. Biotechnol. 105, 4453–4466. doi: 10.1007/s00253-021-11356-1, PMID: 34043082

[ref21] Bayer T. Neave M. J. Alsheikh-Hussain A. Aranda M. Yum L. K. Mincer T.. (2013). The microbiome of the Red Sea coral *Stylophora pistillata* is dominated by tissue-associated Endozoicomonas bacteria. Appl. Environ. Microbiol. 79, 4759–4762. doi: 10.1128/AEM.00695-13, PMID: 23709513 PMC3719505

[ref22] Bayona L. M. de Voogd N. J. Choi Y. H. (2022). Metabolomics on the study of marine organisms. Metabolom. Off. J. Metabol. Soc. 18:17. 10.1007/s11306-022-01874-y PMC8891194 35235054

[ref23] Bhanot A. Sharma R. Noolvi M. N. (2011). Natural sources as potential anti-cancer agents: a review. Int. J. Phytomed. 3, 9–26.

[ref24] Blunt J. W. Carroll A. R. Copp B. R. Davis R. A. Keyzers R. A. Prinsep M. R. (2018). Marine natural products. Nat. Prod. Rep. 35, 8–53. doi: 10.1039/C7NP00052A 29335692

[ref25] Boopathy N. S. Kathiresan K. (2010). Anticancer drugs from marine flora: an overview. J. Oncol. 2010, 1–18. doi: 10.1155/2010/214186, PMID: 21461373 PMC3065217

[ref26] Brumley D. Spencer K. A. Gunasekera S. P. Sauvage T. Biggs J. Paul V. J.. (2018). Isolation and characterization of Anaephenes A–C, Alkylphenols from a filamentous cyanobacterium (Hormoscillasp., Oscillatoriales). J. Nat. Prod. 81, 2716–2721. doi: 10.1021/acs.jnatprod.8b00650, PMID: 30489078 PMC7315913

[ref27] Bunbamrung N. Intaraudom C. Dramae A. Komwijit S. Laorob T. Khamsaeng S.. (2020). Antimicrobial, antimalarial and anticholinesterase substances from the marine-derived fungus aspergillus terreus BCC51799. Tetrahedron 76:131496. doi: 10.1016/j.tet.2020.131496

[ref28] ClinicalTrials.gov. (2023a). Available at: https://clinicaltrials.gov/NCT00003677.

[ref29] ClinicalTrials.gov. (2023b). AvailPable at: https://clinicaltrials.gov/NCT01669252.

[ref30] ClinicalTrials.gov. (2023c). Avaialble at: https://clinicaltrials.gov/NCT00884845.

[ref31] Carroll A. R. Copp B. R. Davis R. A. Keyzers R. A. (2023). Prinsepf marine natural products. Nat. Prod. Rep. 40, 275–325. doi: 10.1039/D2NP00083K 36786022

[ref32] Che Q. Qiao L. Han X. Liu Y. Wang W. Gu Q.. (2018). Anthranosides A–C, anthranilate derivatives from a sponge-DerivedStreptomycessp. CMN-62. Org. Lett. 20, 5466–5469. doi: 10.1021/acs.orglett.8b02382 30106304

[ref33] Cho E. Kwon O.-S. Chung B. Lee J. Sun J. Shin J.. (2020). Antibacterial activity of Chromomycins from a marine-derived *Streptomyces microflavus*. Mar. Drugs 18:522. doi: 10.3390/md18100522 33096696 PMC7588889

[ref34] Chung D. Nguyen H. Yu N. H. Yu W.-J. Kwon Y. M. Bae S. S.. (2023). *In vitro* and *in vivo* antimicrobial activity of the fungal metabolite toluquinol against phytopathogenic bacteria. Front. Microbiol. 14:1221865. doi: 10.3389/fmicb.2023.1221865, PMID: 37583517 PMC10424571

[ref35] Contreras-Castro L. Martinez-Garcia S. Cancino-Diaz J. C. Maldonado L. A. Hernandez-Guerrero C. J. Martínez-Díaz S. F.. (2020). Inhibits the growth of emerging bacterial pathogens and other multi-drug-resistant Bacteria. Pol. J. Microbiol. 69, 321–330. doi: 10.33073/pjm-2020-035 33574861 PMC7810121

[ref36] DrugBank Online. (2023a). AVailable at: http://www.drugbank.ca/DB05109.

[ref37] DrugBank Online. (2023b). Available at: http://www.drugbank.ca/DB08871

[ref38] DrugBank Online. (2023c). Available at: http://www.drugbank.ca/DB05158

[ref39] da Silva A. B. Pinto F. C. Silveira E. R. Costa-Lotufo L. V. Costa W. S. Ayala A. P.. (2019). 4-Hydroxy-pyran-2-one and 3-hydroxy-N-methyl-2-oxindole derivatives of *Salinispora arenicola* from Brazilian marine sediments. Fitoterapia 138:104357. doi: 10.1016/j.fitote.2019.104357 31521701

[ref40] Dadgostar P. (2019). Antimicrobial resistance: implications and costs. Infect. Drug Resist. 12, 3903–3910. doi: 10.2147/IDR.S234610, PMID: 31908502 PMC6929930

[ref41] Dang H. Lovell C. R. (2016). Microbial surface colonization and biofilm development in marine environments. Microbiol. Mol. Biol. Rev. 80, 91–138. doi: 10.1128/MMBR.00037-15, PMID: 26700108 PMC4711185

[ref42] De Carvalho C. C. C. R. (2018). Marine biofilms: a successful microbial strategy with economic implications. Front. Mar. Sci. 5:5. doi: 10.3389/fmars.2018.00126

[ref43] de Sá J. D. M. Kumla D. Dethoup T. Kijjoa A. (2022). Bioactive compounds from terrestrial and marine-derived Fungi of the genus Neosartorya. Molecules 27:2351. doi: 10.3390/molecules27072351 35408769 PMC9000665

[ref44] Deshmukh S. K. Prakash V. Ranjan N. (2018). Marine Fungi: a source of potential anticancer compounds. Front. Microbiol. 8:2536. doi: 10.3389/fmicb.2017.02536, PMID: 29354097 PMC5760561

[ref45] Dobretsov S. Qian P.-Y. (2006). Facilitation and inhibition of larval attachment of the bryozoan *Bugula neritina* in association with mono-species and multi-species biofilms. J. Exp. Mar. Biol. Ecol. 333, 263–274. doi: 10.1016/j.jembe.2006.01.019

[ref46] Domínguez-Oliva A. Hernández-Ávalos I. Martínez-Burnes J. Olmos-Hernández A. Verduzco-Mendoza A. Mota-Rojas D. (2023). The importance of animal models in biomedical research: current insights and applications. Animals 13:1223. doi: 10.3390/ani13071223, PMID: 37048478 PMC10093480

[ref47] Dyshlovoy S. A. Honecker F. (2018). Marine compounds and cancer: 2017 updates. Mar. Drugs 16:41. doi: 10.3390/md16020041 29364147 PMC5852469

[ref48] Elbandy M. (2023). Anti-inflammatory effects of marine bioactive compounds and their potential as functional food ingredients in the prevention and treatment of Neuroinflammatory disorders. Molecules 28:2. doi: 10.3390/molecules28010002 PMC9822486 36615197

[ref49] El-Hossary E. M. Cheng C. Hamed M. M. Hamed A. N. E.-S. Ohlsen K. Hentschel U.. (2017). Antifungal potential of marine natural products. Eur. J. Med. Chem. 126, 631–651. doi: 10.1016/j.ejmech.2016.11.022 27936443

[ref50] Encarnação T. Pais A. A. Campos M. G. Burrows H. D. (2015). Cyanobacteria and microalgae: a renewable source of bioactive compounds and other chemicals. Sci. Prog. 98, 145–168. doi: 10.3184/003685015X14298590596266, PMID: 26288917 PMC10365369

[ref51] Fahmy N. M. Abdel-Tawab A. M. (2021). Isolation and characterization of marine sponge–associated Streptomyces sp. NMF6 strain producing secondary metabolite (s) possessing antimicrobial, antioxidant, anticancer, and antiviral activities. J. Genet. Eng. Biotechnol. 19, 1–14. doi: 10.1186/s43141-021-00203-5 34264405 PMC8281025

[ref52] Fijan S. Kocbek P. Steyer A. Vodiˇcar P. M. Strauss M. (2022). The antimicrobial effect of various single-strain and multi-strain probiotics, dietary supplements or other beneficial microbes against common clinical wound pathogens. Microorganisms 10:2518. doi: 10.3390/microorganisms10122518, PMID: 36557771 PMC9781324

[ref53] Flemming H. C. Wingender J. (2010). The biofilm matrix. Nat. Rev. Microbiol. 8, 623–633. doi: 10.1038/nrmicro2415 20676145

[ref54] Gao C. H. Tian X. P. Qi S. H. Luo X. M. Wang P. Zhang S. (2010). Antibacterial and antilarval compounds from marine gorgonian-associated bacterium *Bacillus amyloliquefaciens* SCSIO 00856. J. Antibiot. 63, 191–193. doi: 10.1038/ja.2010.7 20150929

[ref55] Gao G. Wang Y. Hua H. Li D. Tang C. (2021). Marine antitumor peptide Dolastatin 10: biological activity, structural modification and synthetic chemistry. Mar. Drugs 19:363. doi: 10.3390/md19070363 34202685 PMC8303260

[ref56] Giddings L. A. Newman D. J. (2022). Extremophilic Fungi from marine environments: underexplored sources of antitumor, anti-infective and other biologically active agents. Mar. Drugs 20:62. doi: 10.3390/md20010062 35049917 PMC8781577

[ref57] Gladfelter A. S. James T. Y. Amend A. S. (2019). Marine fungi. Curr. Biol. 29, R191–R195. doi: 10.1016/j.cub.2019.02.009 30889385

[ref58] Gui C. Zhang S. Zhu X. Ding W. Huang H. Gu Y.-C.. (2017). Antimicrobial spirotetronate metabolites from marine-derived Micromonospora harpali SCSIO GJ089. J. Nat. Prod. 80, 1594–1603. doi: 10.1021/acs.jnatprod.7b00176, PMID: 28489382

[ref59] Hafez Ghoran S. Taktaz F. Sousa E. Fernandes C. Kijjoa A. (2023). Peptides from marine-derived Fungi: chemistry and biological activities. Mar. Drugs 21:510. doi: 10.3390/md21100510, PMID: 37888445 PMC10608792

[ref60] Hall-Stoodley L. Costerton J. W. Stoodley P. (2004). Bacterial biofilms: from the natural environment to infectious diseases. Nat. Rev. Microbiol. 2, 95–108. doi: 10.1038/nrmicro821 15040259

[ref61] Hanif N. Murni A. Tanaka C. Tanaka J. (2019). Marine natural products from Indonesian waters. Mar. Drugs 17:364. doi: 10.3390/md17060364 31248122 PMC6627775

[ref62] Hengzhuang W. Wu H. Ciofu O. Song Z. Hoiby N. (2011). Pharmacokinetics/pharmacodynamics of colistin and imipenem on mucoid and nonmucoid *Pseudomonas aeruginosa* biofilms. Antimicrob. Agents Chemother. 55, 4469–4474. doi: 10.1128/AAC.00126-11, PMID: 21670181 PMC3165294

[ref63] Huang H. (2023). Bacteroidetes. Encyclopedia. Available at: https://encyclopedia.pub/entry/32508 (Accessed: 06 November 2023).

[ref64] Islam S. Farjana M. Uddin M. R. Akter S. Jabin A. Nafisa H. T.. (2022). Molecular identification, characterization, and antagonistic activity profiling of *Bacillus cereus* LOCK 1002 along with the in-silico analysis of its presumptive bacteriocins. J. Adv. Vet. Anim. Res. 9, 663–675. doi: 10.5455/javar.2022.i635, PMID: 36714520 PMC9868795

[ref65] Jahromi S. T. Barzkar N. (2018a). Future direction in marine bacterial agarases for industrial applications. Appl. Microbiol. Biotechnol. 102, 6847–6863. doi: 10.1007/s00253-018-9156-5, PMID: 29909571

[ref66] Jahromi S. T. Barzkar N. (2018b). Marine bacterial chitinase as sources of energy, eco-friendly agent, and industrial biocatalyst. Int. J. Biol. Macromol. 120, 2147–2154. doi: 10.1016/j.ijbiomac.2018.09.083, PMID: 30223053

[ref67] Jha R. K. Zi-rong X. (2004). Biomedical compounds from marine organisms. Mar. Drugs 2, 123–146. doi: 10.3390/md203123

[ref68] Ji X. Guo J. Liu Y. Lu A. Wang Z. Li Y.. (2018). Marine-natural-product development: first discovery of nortopsentin alkaloids as novel antiviral, anti-phytopathogenic-fungus, and insecticidal agents. J. Agric. Food Chem. 66, 4062–4072. doi: 10.1021/acs.jafc.8b00507, PMID: 29630371

[ref69] Jiménez C. (2018). Marine natural products in medicinal chemistry. ACS Med. Chem. Lett. 9, 959–961. doi: 10.1021/acsmedchemlett.8b00368 30344898 PMC6187399

[ref70] Karim M. R. U. Harunari E. Oku N. Akasaka K. Igarashi Y. (2020). Bulbimidazoles A–C, antimicrobial and cytotoxic Alkanoyl Imidazoles from a marine Gammaproteobacterium MicrobulbiferSpecies. J. Nat. Prod. 83, 1295–1299. doi: 10.1021/acs.jnatprod.0c00082, PMID: 32191468

[ref71] Kariyazono R. Ito S. Osanai T. (2022). Carbon metabolism of great biotechnological interest: Metabolic engineering and synthetic biology of cyanobacteria. Cyanobact. Physiol., 189–200. doi: 10.1016/B978-0-323-96106-6.00003-4

[ref72] Karthikeyan A. Joseph A. Nair B. G. (2022). Promising bioactive compounds from the marine environment and their potential effects on various diseases. J. Genet. Eng. Biotechnol. 20:14. doi: 10.1186/s43141-021-00290-4 35080679 PMC8790952

[ref73] Khalifa S. A. Elias N. Farag M. A. Chen L. Saeed A. Hegazy M.-E. F.. (2019). Marine natural products: a source of novel anticancer drugs. Mar. Drugs 17:491. doi: 10.3390/md17090491 31443597 PMC6780632

[ref74] Khotimchenko Yu. S., Biologically active substances from marine aquatic organisms - a source of new pharmaceutical substances and drugs. TMG. (2010). 2. Avaialble at: https://cyberleninka.ru/article/n/biologicheski-aktivnye-veschestva-iz-morskih-gidrobiontov-istochnik-novyh-farmatsevticheskih-substantsiy-i-lekarstv (Accessed: 21 November, 2023).

[ref75] Kuo J. Yang Y.-T. Lu M.-C. Wong T.-Y. Sung P.-J. Huang Y.-S. (2019). Antimicrobial activity and diversity of bacteria associated with Taiwanese marine sponge Theonella swinhoei. Ann. Microbiol. 69, 253–265. doi: 10.1007/s13213-018-1414-3

[ref76] Lackner G. Peters E. E. Helfrich E. J. Piel J. (2017). Insights into the lifestyle of uncultured bacterial natural product factories associated with marine sponges. Proc. Natl. Acad. Sci. U. S. A 114, E347–E356. doi: 10.1073/pnas.1616234114 28049838 PMC5255618

[ref77] Li Z. Wang Y. Li X. Lin Z. Lin Y. Srinivasan R.. (2019). The characteristics of antibiotic resistance and phenotypes in 29 outer-membrane protein mutant strains in *Aeromonas hydrophila*. Environ. Microbiol. 21, 4614–4628. doi: 10.1111/1462-2920.14761 31355499

[ref78] Liu K. Ding H. Yu Y. Chen B. (2019). A cold-adapted chitinase-producing bacterium from Antarctica and its potential in biocontrol of plant pathogenic fungi. Mar. Drugs 17:695. doi: 10.3390/md17120695 31835449 PMC6950295

[ref79] Long L. Wang R. Chiang H. Y. Ding W. Li Y. X. Chen F.. (2021). Discovery of Antibiofilm activity of Elasnin against marine biofilms and its application in the marine antifouling coatings. Mar. Drugs 19:19. doi: 10.3390/md19010019, PMID: 33466541 PMC7824865

[ref80] Makarieva T. N. Silchenko A. S. Kicha A. A. Lyakhova E. G. Kolesnikova S. A. Shubina L. K.. (2014). Search and isolation of new natural compounds from marine invertebrates, studies of their structures and biological activities. Bull. Far East. Branch Russ. Acad. Sci. 1

[ref81] Martyniuk A. O. Skrotska O. I. Shevchuk T. A. Pirog T. P. (2020). Practically valuable metabolites of marine microorganisms. Biotechnol. Acta:3.

[ref82] Maslin M. Gaertner-Mazouni N. Debitus C. Joy N. Ho R. (2021). Marine sponge aquaculture towards drug development: an ongoing history of technical, ecological, chemical considerations and challenges. Aquacult. Rep. 21:100813. doi: 10.1016/j.aqrep.2021.100813

[ref83] Masschelein J. Jenner M. Challis G. L. (2017). Antibiotics from gram-negative bacteria: a comprehensive overview and selected biosynthetic highlights. Nat. Prod. Rep. 34, 712–783. doi: 10.1039/C7NP00010C, PMID: 28650032

[ref84] McCarthy P. J. Roberts B. F. Carbonell A. Roberts J. Wright A. E. Chakrabarti D. (2019). Marine microbiome as a source of antimalarials. Trop. Med. Infect. Dis. 4:103. doi: 10.3390/tropicalmed4030103 31337089 PMC6789460

[ref85] Meyer V. Basenko E. Y. Benz J. P. (2020). Growing a circular economy with fungal biotechnology: a white paper. Biol. Biotechnol. 7:5. doi: 10.1186/s40694-020-00095-z PMC7140391 32280481

[ref86] Miksch S. Meiners M. Meyerdierks A. Probandt D. Wegener G. Titschack J. (2021). Bacterial communities in temperate and polar coastal sands are seasonally stable. ISME Commun. 1, 1–11. doi: 10.1038/s43705-021-00028-w 36739458 PMC9723697

[ref87] Mohie T. El-dien R. Mahmoud B. K. Abdelwahab M. F. (2023). Paralemnalia thyrsoides-associated fungi: phylogenetic diversity, cytotoxic potential, metabolomic profiling and docking analysis. BMC Microbiol. 23:308. doi: 10.1186/s12866-023-03045-y 37884900 PMC10601334

[ref88] Mulani M. S. Kamble E. E. Kumkar S. N. Tawre M. S. Pardesi K. R. (2019). Emerging strategies to combat ESKAPE pathogens in the era of antimicrobial resistance: a review. Front. Microbiol. 10:539. doi: 10.3389/fmicb.2019.00539, PMID: 30988669 PMC6452778

[ref89] Naeem A. Hu P. Yang M. Zhang J. Liu Y. Zhu W.. (2022). Natural products as anticancer agents: current status and future perspectives. Molecules 27:8367. doi: 10.3390/molecules27238367, PMID: 36500466 PMC9737905

[ref90] Newman D. J. Cragg G. M. (2004). Advanced preclinical and clinical trials of natural products and related compounds from marine sources. Curr. Med. Chem. 11, 1693–1713. doi: 10.2174/0929867043364982 15279577

[ref91] Newman D. J. Cragg G. M. (2020). Natural products as sources of new drugs over the nearly four decades from 01/1981 to 09/2019. J. Nat. Prod. 83, 770–803. doi: 10.1021/acs.jnatprod.9b01285, PMID: 32162523

[ref92] Ng Z. J. Zarin M. A. Lee C. K. Tan J. S. (2020). Application of bacteriocins in food preservation and infectious disease treatment for humans and livestock: a review. RSC Adv. 10, 38937–38964. doi: 10.1039/D0RA06161A 35518417 PMC9057404

[ref93] Ngamcharungchit C. Chaimusik N. Panbangred W. Euanorasetr J. Intra B. (2023). Bioactive metabolites from terrestrial and marine Actinomycetes. Molecules 28:5915. doi: 10.3390/molecules28155915 37570885 PMC10421486

[ref94] Nicoletti R. Salvatore M. M. Andolfi A. (2018). Secondary metabolites of mangrove-associated strains of Talaromyces. Mar. Drugs 16:12. doi: 10.3390/md16010012 29316607 PMC5793060

[ref95] Nielsen J. C. Grijseels S. Prigent S. Ji B. Dainat J. Nielsen K. F.. (2017). Global analysis of biosynthetic gene clusters reveals vast potential of secondary metabolite production in Penicillium species. Nat. Microbiol. 2, 1–9. doi: 10.1038/nmicrobiol.2017.44 28368369

[ref96] Nobili S. Lippi D. Witort E. Donnini M. Bausi L. Mini E.. (2009). Natural compounds for cancer treatment and prevention. Pharmacol. Res. 59, 365–378. doi: 10.1016/j.phrs.2009.01.017 19429468

[ref97] Nsanzabana C. (2019). Resistance to artemisinin combination therapies (ACTs): do not forget the partner drug! Trop. Med. Infect. Dis. 4:26. doi: 10.3390/tropicalmed4010026 30717149 PMC6473515

[ref98] Nugraha A. S. Firli L. N. Rani D. M. (2023). Indonesian marine and its medicinal contribution. Nat. Prod. Rep. 13:38. doi: 10.1007/s13659-023-00403-1 PMC10579215 37843645

[ref99] O’Toole G. Kaplan H. B. Kolter R. (2000). Biofilm formation as microbial development. Annu. Rev. Microbiol. 54, 49–79. doi: 10.1146/annurev.micro.54.1.49 11018124

[ref100] Obaid H. M. Sale S. S. Boundenga L. (2023). Pharmaceutical activity of a synthetic heterocyclic (C15H12N5OCl) compound on Entamoeba histolytica and *Giardia lamblia*. Russ. J. Infect. Immun. 13, 119–126. doi: 10.15789/2220-7619-PAO-2024

[ref101] Ohno H. Saheki T. Awaya J. Nakagawa A. Omura S. (1978). Isolation and characterization of elasnin, a new human granulocyte elastase inhibitor produced by a strain of Streptomyces. J. Antibiot. 31, 1116–1123. doi: 10.7164/antibiotics.31.1116, PMID: 721707

[ref102] Packiavathy I. A. S. V. Kannappan A. Thiyagarajan S. Srinivasan R. Jeyapragash D. Paul J. B. J.. (2021). AHL-lactonase producing Psychrobacter sp. from Palk Bay sediment mitigates quorum sensing-mediated virulence production in gram negative bacterial pathogens. Front. Microbiol. 12:634593. doi: 10.3389/fmicb.2021.634593, PMID: 33935995 PMC8079732

[ref103] Pedrosa R. Gaudêncio P. Vasconcelos S. (2020). XVI international symposium on marine natural products| XI European conference on marine natural products. Mar. Drugs 18:40. 31935809 10.3390/md18010040 PMC7024214

[ref104] Perera R. M. T. D. Herath K. H. I. N. M. Sanjeewa K. K. A. Jayawardena T. U. (2023). Recent reports on bioactive compounds from marine Cyanobacteria in relation to human health applications. Life (Basel, Switzerland) 13:1411. doi: 10.3390/life13061411 37374193 PMC10301180

[ref105] Pike R. E. Haltli B. Kerr R. G. (2013). Description of Endozoicomonas euniceicola sp. nov. and Endozoicomonas gorgoniicola sp. nov., bacteria isolated from the octocorals Eunicea fusca and Plexaura sp., and an emended description of the genus Endozoicomonas. Int. J. Syst. Evol. Microbiol. 63, 4294–4302. doi: 10.1099/ijs.0.051490-0 23832969

[ref106] Plaza G. Achal V. (2020). Biosurfactants: eco-friendly and innovative biocides against biocorrosion. Int. J. Mol. Sci. 21:2152. doi: 10.3390/ijms21062152, PMID: 32245097 PMC7139319

[ref107] Poli A. Finore I. Romano I. Gioiello A. Lama L. Nicolaus B. (2017). Microbial diversity in extreme marine habitats and their biomolecules. Microorganisms 5:25. doi: 10.3390/microorganisms5020025 28509857 PMC5488096

[ref108] Premarathna A. D. Ahmed T. A. E. Kulshreshtha G. Humayun S. Darko C. N. S. Rjabovs V.. (2024). Polysaccharides from red seaweeds: effect of extraction methods on physicochemical characteristics and antioxidant activities. Food Hydrocoll. 147:109307. doi: 10.1016/j.foodhyd.2023.109307

[ref109] Puttaswamygowda G. H. Olakkaran S. Antony A. Purayil A. K. (2019). Present status and future perspectives of marine Actinobacterial metabolites. Recent Dev. Appl. Microbiol. Biochem., 307–319. doi: 10.1016/B978-0-12-816328-3.00022-2

[ref110] Qian P. Y. Lau S. C. Dahms H. U. Dobretsov S. Harder T. (2007). Marine biofilms as mediators of colonization by marine macroorganisms: implications for antifouling and aquaculture. Mar. Biotechnol. 9, 399–410. doi: 10.1007/s10126-007-9001-9, PMID: 17497196

[ref111] Ribeiro I. Antunes J. T. Alexandrino D. A. M. Tomasino M. P. Almeida E. Hilário A.. (2023). Actinobacteria from Arctic and Atlantic deep-sea sediments-biodiversity and bioactive potential. Front. Microbiol. 14:1158441. doi: 10.3389/fmicb.2023.1158441, PMID: 37065153 PMC10100589

[ref112] Robles-Bañuelos B. Durán-Riveroll L. M. Rangel-López E. Pérez-López H. I. González-Maya L. (2022). Marine Cyanobacteria as sources of Lead anticancer compounds: a review of families of metabolites with cytotoxic, Antiproliferative, and antineoplastic effects. Molecules 27:4814. doi: 10.3390/molecules27154814 35956762 PMC9369884

[ref113] Rocha-Martin J. Harrington C. Dobson A. D. O’Gara F. (2014). Emerging strategies and integrated systems microbiology technologies for biodiscovery of marine bioactive compounds. Mar. Drugs 12, 3516–3559. doi: 10.3390/md12063516, PMID: 24918453 PMC4071589

[ref114] Rommasi F. (2022). Bacterial-based methods for Cancer treatment: what we know and where we are. Oncol. Ther. 10, 23–54. doi: 10.1007/s40487-021-00177-x, PMID: 34780046 PMC9098760

[ref115] Rua C. P. Trindade-Silva A. E. Appolinario L. R. Venas T. M. Garcia G. D. Carvalho L. S.. (2014). Diversity and antimicrobial potential of culturable heterotrophic bacteria associated with the endemic marine sponge Arenosclera brasiliensis. Peer J 2:e419. doi: 10.7717/peerj.419, PMID: 25024903 PMC4081303

[ref116] Ruginescu R. Enache M. Popescu O. Gomoiu I. Cojoc R. Microorgan Batrinescu-Moteau C.. (2022). Characterization of some salt-tolerant bacterial hydrolases with potential utility in cultural heritage bio-cleaning. Nat. Prod. Rep. 10:644. doi: 10.3390/microorganisms10030644 PMC8949325 35336219

[ref117] Ruiz-Gil T. Acuña J. J. Fujiyoshi S. Tanaka D. Noda J. Maruyama F.. (2020). Airborne bacterial communities of outdoor environments and their associated influencing factors. Environ. Int. 145:106156. doi: 10.1016/j.envint.2020.106156, PMID: 33039877

[ref118] Rungprom W. Siwu E. R. Lambert L. K. Dechsakulwatana C. Barden M. C. Kokpol U.. (2008). Cyclic tetrapeptides from marine bacteria associated with the seaweed Diginea sp. and the sponge *Halisarca ectofibrosa*. Tetrahedron 64, 3147–3152. doi: 10.1016/j.tet.2008.01.089

[ref119] Sannino F. Parrilli E. Apuzzo G. A. de Pascale D. Tedesco P. Maida I.. (2017). *Pseudoalteromonas haloplanktis* produces methylamine, a volatile compound active against *Burkholderia cepacia* complex strains. New Biotechnol. 35, 13–18. doi: 10.1016/j.nbt.2016.10.009, PMID: 27989956

[ref120] Saurav K. Costantino V. Venturi V. Steindler L. (2017). Quorum sensing inhibitors from the sea discovered using bacterial N-acyl-homoserine lactone-based biosensors. Mar. Drugs 15:53. doi: 10.3390/md15030053 28241461 PMC5367010

[ref121] Schofield M. M. Jain S. Porat D. Dick G. J. Sherman D. H. (2015). Identification and analysis of the bacterial endosymbiont specialized for production of the chemotherapeutic natural product ET-743. Environ. Microbiol. 17, 3964–3975. doi: 10.1111/1462-2920.12908, PMID: 26013440 PMC4618771

[ref122] Schultz F. Anywar G. Tang H. Chassagne F. Lyles J. T. Garbe L.-A.. (2020). Targeting ESKAPE pathogens with anti-infective medicinal plants from the greater Mpigi region in Uganda. Sci. Rep. 10, 1–19. doi: 10.1038/s41598-020-67572-8 32686689 PMC7371678

[ref123] Shafiekhani M. Shekari Z. Boorboor A. (2022). Bacterial and fungal co-infections with SARS-CoV-2 in solid organ recipients: a retrospective study. Virol. J. 19:35. doi: 10.1186/s12985-022-01763-9, PMID: 35246169 PMC8894563

[ref124] Shahidi F. Santhiravel S. (2022). Novel marine bioactives: application in functional foods, nutraceuticals, and pharmaceuticals. J. Food Bioact. 19:19. doi: 10.31665/JFB.2022.18316

[ref125] Srilekha V. Krishna G. Seshasrinivas V. Charya M. A. S. (2017). Antibacterial and anti-inflammatory activities of marine Brevibacterium sp. Res. Pharm. Sci. 12, 283–289. doi: 10.4103/1735-5362.212045, PMID: 28855939 PMC5566002

[ref126] Srinivasan R. Kannappan A. Shi C. Lin X. (2021). Marine bacterial secondary metabolites: a treasure house for structurally unique and effective antimicrobial compounds. Mar. Drugs 19:530. doi: 10.3390/md19100530 34677431 PMC8539464

[ref127] Stincone P. Brandelli A. (2020). Marine bacteria as source of antimicrobial compounds. Crit. Rev. Biotechnol. 40, 306–319. doi: 10.1080/07388551.2019.1710457 31992085

[ref128] Stonik V. A. Makarieva T. N. Shubina L. K. (2020). Antibiotics from marine Bacteria. Biochem. Mosc. 85, 1362–1373. doi: 10.1134/S0006297920110073, PMID: 33280579

[ref129] Sun W. Wu W. Liu X. Zaleta-Pinet D. A. Clark B. R. (2019). Bioactive compounds isolated from marine-derived microbes in China: 2009–2018. Mar. Drugs 17:339. doi: 10.3390/md17060339, PMID: 31174259 PMC6628246

[ref130] Tajuddeen N. Van Heerden F. R. (2019). Antiplasmodial natural products: an update. Malar. J. 18:404. doi: 10.1186/s12936-019-3026-1, PMID: 31805944 PMC6896759

[ref131] Tan L. T. (2007). Bioactive natural products from marine cyanobacteria for drug discovery. Phytochemistry 68, 954–979. doi: 10.1016/j.phytochem.2007.01.012 17336349

[ref132] Tan L. T. (2023). Impact of marine chemical ecology research on the discovery and development of new pharmaceuticals. Mar. Drugs 21:174. doi: 10.3390/md21030174, PMID: 36976223 PMC10055925

[ref133] Trial.gov, C. (2023) 27 May 2023.

[ref134] Uzair B. Bano A. Niazi M. B. K. Khan F. Mujtaba G. (2018). In vitro antifungal activity of 9, 10-dihydrophenanthrene-2-carboxylic acid isolated from a marine bacterium: *Pseudomonas putida*. Pak. J. Pharm. Sci. 31, 2733–2736. 30587487

[ref135] Wang W. Park K. H. Lee J. Oh E. Park C. Kang E.. (2020). A new Thiopeptide antibiotic, Micrococcin P3, from a marine-derived strain of the bacterium *Bacillus stratosphericus*. Molecules. Basel, Switzerland 25:4383. doi: 10.3390/molecules25194383 PMC7582574 32987657

[ref136] Wefky S. Hassan M. Antibacterial (2018). Anticoagulant and anti-inflammatory activities of marine *Bacillus cereus* S1. J. Pure Appl. Microbiol. 10, 2593–2606. doi: 10.22207/JPAM.10.4.15

[ref137] White M. C. Holman D. M. Boehm J. E. Peipins L. A. Grossman M. Henley S. J. (2014). Age and cancer risk: a potentially modifiable relationship. Am. J. Prev. Med. 46, S7–S15. doi: 10.1016/j.amepre.2013.10.029, PMID: 24512933 PMC4544764

[ref138] WHO (2020). World malaria report: 20 years of global progress and challenges. World malaria report 2020: 20 years of global progress and challenges 2020, 299

[ref139] Xu P. Ding L. Wei J. Li Q. Gui M. He X.. (2020). A new aquatic pathogen inhibitor produced by the marine fungus aspergillus sp. LS116. Aquaculture 520:734670. doi: 10.1016/j.aquaculture.2019.734670

[ref140] Xu J.-L. Liu H.-X. Chen Y.-C. Tan H.-B. Guo H. Xu L.-Q.. (2018). Highly substituted benzophenone aldehydes and Eremophilane derivatives from the Deep-Sea derived fungus Phomopsis lithocarpus FS508. Mar. Drugs 16:329. doi: 10.3390/md16090329 30208615 PMC6165036

[ref141] Yang Y. Kessler M. G. C. Marchán-Rivadeneira M. R. Han Y. (2023). Combating antimicrobial resistance in the post-genomic era: rapid antibiotic discovery. Molecules 28:4183. doi: 10.3390/molecules28104183, PMID: 37241928 PMC10221052

[ref142] Yasir M. (2018). Analysis of bacterial communities and characterization of antimicrobial strains from cave microbiota. Braz. J. Microbiol. 49, 248–257. doi: 10.1016/j.bjm.2017.08.005, PMID: 29108974 PMC5913830

[ref143] Ye X. Anjum K. Song T. Wang W. Liang Y. Chen M.. (2017). Antiproliferative cyclodepsipeptides from the marine actinomycete Streptomyces sp. P11-23B downregulating the tumor metabolic enzymes of glycolysis, glutaminolysis, and lipogenesis. Phytochemistry 135, 151–159. doi: 10.1016/j.phytochem.2016.12.010, PMID: 28049552 PMC7111624

[ref144] Zhang Z. Wang P. Chen M. Xie L. Zhang X. Shi Y.. (2023). Antibacterial activity of two new Cassane Diterpenoids from Caesaplinia pulcherrima against *Bacillus cereus* by damage to cell membrane. Int. J. Mol. Sci. 24:4917. 36902346 10.3390/ijms24054917 PMC10003239

[ref145] Zhang B. Wang K. B. Wang W. Bi S. F. Mei Y. N. Deng X. Z.. (2018). Discovery, biosynthesis, and heterologous production of streptoseomycin, an anti-microaerophilic bacteria macrodilactone. Org. Lett. 20, 2967–2971. doi: 10.1021/acs.orglett.8b01006, PMID: 29697266

[ref146] Zhao P. Xue Y. Li J. (2019). Non-lipopeptide fungi-derived peptide antibiotics developed since 2000. Biotechnol. Lett. 41, 651–673. doi: 10.1007/s10529-019-02677-3, PMID: 31020454

[ref147] Zhou W. Liang H. Qin X. Cao D. Zhu X. Ju J.. (2020). The isolation of Pyrroloformamide congeners and characterization of their biosynthetic gene cluster. J. Nat. Prod. 83, 202–209. doi: 10.1021/acs.jnatprod.9b00321, PMID: 32049520 PMC7577424

[ref148] Zong R. Ruan H. Liu C. Fan S. Li J. (2023). Bacteria and bacterial components as natural bio-Nanocarriers for drug and gene delivery Systems in Cancer Therapy. Pharmaceutics 15:2490. doi: 10.3390/pharmaceutics15102490, PMID: 37896250 PMC10610331

